# Comparison of Lifetime of the PVD Coatings in Laboratory Dynamic Impact Test and Industrial Fine Blanking Process

**DOI:** 10.3390/ma13092154

**Published:** 2020-05-06

**Authors:** Josef Daniel, Radek Žemlička, Jan Grossman, Andreas Lümkemann, Peter Tapp, Christian Galamand, Tomáš Fořt

**Affiliations:** 1 Department of New Technologies, Institute of Scientific Instruments of the Czech Academy of Sciences, Královopolská 147, 612 64 Brno, Czech Republic; grossman@isibrno.cz (J.G.); fortt@isibrno.cz (T.F.); 2 Platit AG, Eichholzstrasse 9, CH-2545 Selzach, Switzerland; r.zemlicka@platit.com (R.Ž.); a.luemkemann@platit.com (A.L.); p.tapp@platit.com (P.T.); c.galamand@platit.com (C.G.)

**Keywords:** dynamic impact test, fine blanking, PVD coatings, impact wear

## Abstract

Protective hard PVD coatings are used to improve the endurance of the tools exposed to repeated impact load, e.g., fine blanking punches. During the fine blanking process, a coated punch repeatedly impacts sheet metal. Thus, the coating which protects the punch surface is exposed to the dynamic impact load. On the other hand, the laboratory method of dynamic impact testing is well known and used for the development and optimization of protective coatings. This paper is focused on the comparison of tool life and lifetime of the industrial prepared PVD coatings exposed to repeated dynamic impact load in the industrial fine blanking process and the laboratory dynamic impact testing. Three different types of protective coatings were tested and the results were discussed. It was shown that the lifetime of coated specimens in both the fine blanking and the dynamic impact processes was influenced by similar mechanical properties of the protective coatings. The qualitative comparison shows that the lifetime obtained by the dynamic impact test was the same as the lifetime obtained by the industrial fine blanking process. The laboratory impact test appears to be a suitable alternative for the optimisation and development of protective PVD coatings for punches used in the industrial fine blanking process.

## 1. Introduction

Punches used in material processing, such as industrial fine blanking, are exposed to extreme contact conditions such as high contact pressure or dynamic impact loads [1]. Those conditions lead to considerable wear and decrease the lifespan of the punch tool [2,3]. The lifespan of the punch can be improved in several ways—by an optimization of fine blanking tools to achieve lower stress [4] or by using a suitable metal sheet as a punched material [5,6]. Another well-known way to improve the punching tool lifespan is the application of protective, hard, and wear-resistant physical vapor deposited (PVD) coating [1,4,7,8,9,10,11].

It was shown that, for a high lifetime of protective PVD coatings, an optimal combination of mechanical properties is required, e.g., high hardness [12], low elastic modulus [13], high toughness [1] and good adhesion to the substrate [4]. However, determination and mutual optimization of all of these properties are insufficient for the development of the new protective PVD coatings for punches. Moreover, for the final estimation of a coatings lifetime, it is necessary to test protective coatings under real industrial conditions. Since the industrial fine blanking process is unapproachable for many institutions, a laboratory test of a coating’s lifetime under conditions similar to industrial ones would be helpful. Such a method is offered: the laboratory dynamic impact test.

In dynamic impact testing, a ball indenter repeatedly impacts the same place of surface of the coated specimen [14,15]. Coating failure is caused by dynamic load, which is similar to fine blanking. This work is focused on the comparison of the lifetime of protective PVD coatings under conditions of the industrial fine blanking process and the laboratory dynamic impact test. At first, the fine blanking process and the dynamic impact test were qualitatively compared. Properties of coatings required for a high lifetime (or tool life) obtained from current literature were compared for the industrial fine blanking process and laboratory dynamic impact test. In total, three industrially prepared PVD coatings with different structures and compositions were tested by both methods. The results of the coating’s lifetimes and behavior under dynamic load were compared and discussed.

## 2. Experimental

### 2.1. Sample Preparation and Testing

P/M S390 Microclean high-speed steel (HSS) fine blanking punches made by Feintool Technologie AG (Lyss, Switzerland) were used as a substrate for testing in an industrial fine blanking process. For the elimination of the substrate influence for the comparison of methods, the substrates from the same type of HSS were used for dynamic impact testing. Three industrial nitride-based PVD coatings with different compositions and structures were used—All^4^-eco^®^, AlCrN^3^-tribo^®^, and Super TiN^®^. The coatings were deposited by PLATIT AG (Selzach, Switzerland) using a low-pressure (~10^−2^ mbar) cathodic arc deposition technique on the PLATIT π^411^ coating unit equipped with four rotary cylindrical cathodes. All of the coatings were deposited in the reactive mode by sputtering of metallic targets in a nitrogen atmosphere. The carbo-nitride layers (see below) were prepared using a nitrogen atmosphere with acetylene addition.

Hardness and effective elastic modulus of the tested coatings was measured using MTS Nano Indenter XP equipped by Berkovich indenter (Oak Ridge, TN, USA). Loads of 20, 30, and 40 mN were used. Hardness and effective elastic modulus were calculated according to Oliver–Pharr [16]. Coating thickness was estimated from deposition rate and deposition time and measured using calotester for proving.

**Figure 1:**
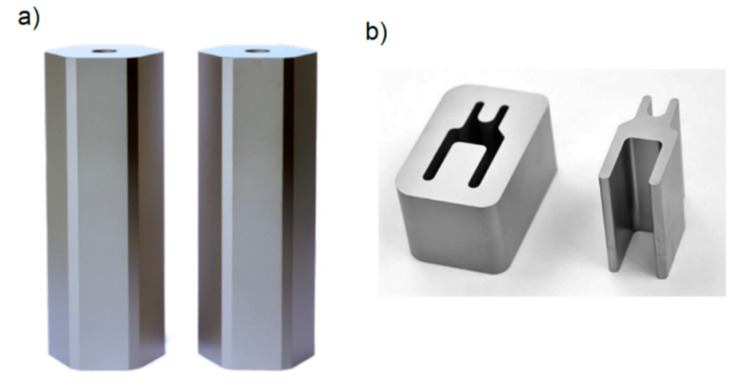
Profiles of the fine-blanking punches. (**a**) hexagonal punch, (**b**) “H-shaped” punch. Source of the picture: Feintool Technologie AG.

The industrial fine blanking tests were carried out using two types of punches. The punch with the hexagonal profile is shown in Figure 1a and the punch with the “H-shape” profile is depicted in Figure 1b. As a work piece material for the fine blanking process, we used two steel sheets. A stainless-steel sheet 1.4509 (thickness of 2.0 mm) was used as a work piece material for the testing with the hexagonal punch and a stainless-steel sheet 1.4301 with (thickness of 2.0 mm) was used as a work piece material for the testing with “H-shaped” punch. Different types of work piece steel sheets were chosen in order to testing on the real materials using in industrial practice. All the industrial fine blanking tests were carried out Feintool Technology AG (Lyss, Switzerland) on the Feintool HFA 4500plus hydraulic fine blanking press with a punch frequency of 1.3 Hz.

**Figure 2:**
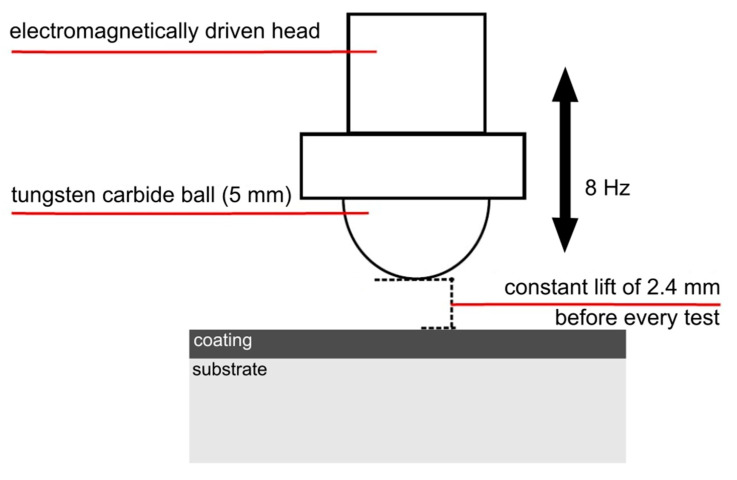
Scheme of the dynamic impact tester.

Laboratory dynamic impact tests were obtained using the electromagnetically driven impact test device developed at the Institute of Scientific Instruments CAS in Brno [17]. This tester is schematically depicted in Figure 2. Impacting was performed by a cemented tungsten carbide indenter (ball-shaped; Ø 5 mm) with surface roughness Ra ≤ 20 nm (ISO 3290 with precision grade 10). To use the well-defined impact load, the lift of the indenter was set to a constant value of 2.4 mm before every testing procedure. The tester impacted the surface of the sample with a repetitive frequency of 8 Hz, the duration of the contact of the indenter with the surface was 1.5 ms.

The impact tester worked with impact loads of 200 N and 600 N. The test was carried out in the range from 1 impact up to 500,000 impacts and every test was repeated three times. Before every test, the indenter ball was turned onto an unworn contact side. Parameters (radius, depth) of the resulted impact craters were estimated using a Talystep profilometer (Taylor Hobson, Leicester, UK). The shape and morphology of the impact craters were analyzed by a confocal microscope Lext OLS 3100 (Olympus, Tokyo, Japan).

### 2.2. Comparison of Industrial Fine Blanking Process and Laboratory Dynamic Impact Test

The industrial fine blanking process is a material processing method that uses reduced clearance between a dynamically loaded punch and die to obtain improved cutting surface quality [4]. During the industrial fine blanking process, the coated punch repeatedly impacts the surface of the work piece material. The punch is exposed to cyclic mechanical and tribological loads, which leads to fatigue and wear [8,18]. Thus, the protective PVD coatings which cover punching tools need to resist extreme conditions, such as a high impact load, high contact wear, or high contact pressures [1,8]. It was shown that such coatings with high fine blanking tool life exhibited optimal combinations of mechanical and tribological properties, such as a high hardness [9,19,20], high H/E ratio [1], good wear resistance [1], good coating adhesion to the substrate [4], and high fracture toughness [1,8,19].

On the other hand, in the laboratory dynamic impact test method, a static specimen coated by protective coating is repeatedly impacted by an impact indenter [14]. The coated specimen is exposed to a combination of cyclic mechanical impacting load and tribological load, which cause the impact wear and fatigue of the coating [17,21,22]. Protective PVD coatings which cover specimens are thus required to cope with high impact loads and high contact pressures [14,23]. It was shown that such coatings need to exhibit an optimal combination of mechanical and tribological properties, such as a high hardness [13,24,25], high H/E ratio [26], good coating adhesion to the substrate [15,21,27], good wear resistance [28], and high fracture toughness [15,29] to reach a high impact lifetime of the coatings.

The most important difference between the industrial fine blanking process and laboratory dynamic impact test is the different stress distribution during loading. In the case of the laboratory dynamic impact test, a ball indentor interacts with a flat surface of the coated specimen. According to the Hertzian quasi-static theory, stress is distributed in hemispherical “core” with the beginning in the point of contact between ball indenter and specimen [30,31]. The tangential component of the stress causes material transport, resulting in creating pile-ups or cracking, depending on coating material properties and its adhesion to the substrate [7,32,33]. In the case of the industrial fine blanking process, the flat-coated punch dynamically interacts with the flat steel sheet [34]. Assuming ideal flat surfaces, stress is distributed perpendicularly to the coated punch surface with the constant value [30]. However, on the edge of punch, distributed stress achieves a different value, and it can be supposed that its tangential component influences coating behaviour [35].

Except for differences in the stress distribution, the lifetime (or tool life) of the protective PVD coatings depends on similar parameters in both the industrial fine blanking process and the laboratory impact test. Additionally, crucial mechanical and tribological properties of coatings are very similar in both processes. Thus, we suppose that the laboratory dynamic impact test is a suitable method for the qualitative determination of the lifetime of protective PVD coatings applied in the industrial fine blanking process. The results obtained by both methods are set and discussed in the next chapter.

## 3. Results

### 3.1. Tested Coatings

**Figure 3:**
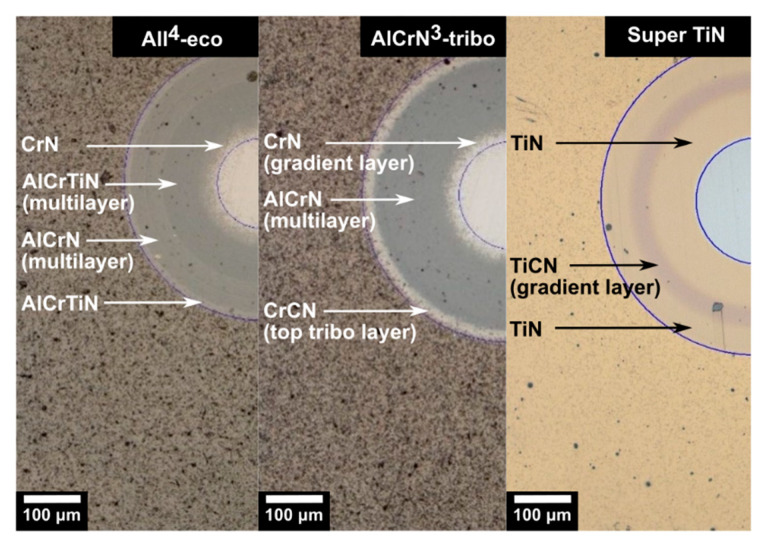
Structure of the tested coatings.

Three PVD coatings, AlCrTi-based nanolayered coating All^4^-eco, AlCr-based nanolayered coating AlCrN^3^-tribo with CrCN top tribo-layer, and Ti-based multilayered coating Super TiN were deposited on the fine blanking punches and the cylindrical HSS substrates. All of the coatings were designed to have the same thickness of ~2.5 µm. The described structure of the coatings on their spherical ball-craters created by a calotester is shown in Figure 3.

Measured hardness and elastic modulus were completed with the calculation of the H/E ratio associated with elastic strain to failure [13] and H^3^/E^2^ ratio related to the plastic deformation resistance [36] and coating toughness [37]. The coefficient of friction (CoF) was measured using a tribometer with a 100Cr6 bearing steel pin. The normal load was set to 2 N and speed 3 cm·s^−1^. An overview of the coating mechanical properties and CoF is summarized in Table 1.

Adhesion of the tested coatings was determined using the adhesion Rockwell-C test and the scratch test. In the case of scratch measurements, the parameter of L_C1_ denotes the load when the first cracking occurs and the parameter of L_C2_ denotes the load where the first delamination on the scratch path occurs. All of the coatings exhibited L_C3_, the load when complete delamination occurs, higher than 100 N. The exact value of L_C3_ was not measured. All data are summarized in Table 2.

### 3.2. Industrial Fine Blanking Tests

**Figure 4:**
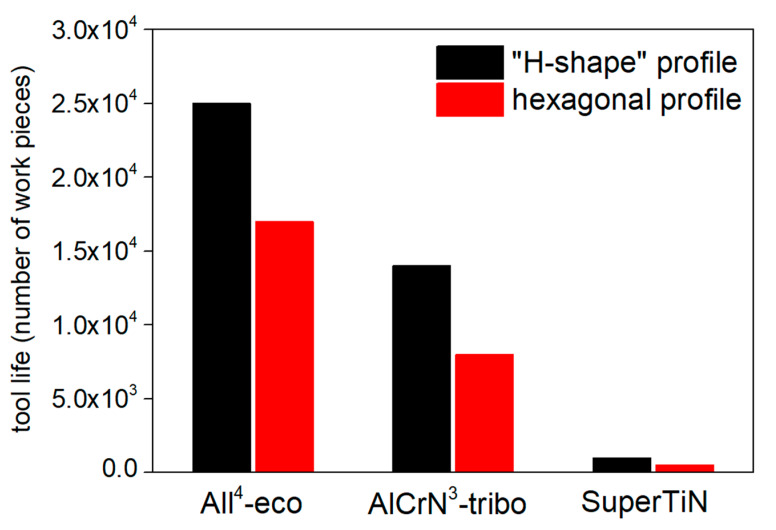
Tool life of the PVD coatings in number of work pieces tested by the industrial fine-blanking process.

The fine blanking tests were carried out under a pressure of 0.44 GPa. The tool life of the PVD coating was evaluated as the number of work pieces without burrs on their edges prepared by a coated punch. The resulting tool life of the coatings tested by the fine blanking process is shown in Figure 4. Generally, the coatings on the “H-shaped” punch exhibited higher tool life than the same coatings on the hexagonally shaped punch. Coating All^4^-eco exhibited the best tool life, 25,000 work pieces in case of the “H-shaped” punch, and 17,000 work pieces in case of the hexagonal profile punch. On the other hand, the tool life of the Super TiN coating was the lowest—1000 work pieces for the “H-shaped” punch and 500 work pieces for the hexagonal profile punch.

As was shown by Lind et al., the tool life of the PVD coatings tested on the industrial fine blanking machine increased with increasing H/E ratio and with lowering elastic modulus, which led to lower stress and thus higher tool life [1]. In our work, the tool life of the tested coatings increased with the increasing H/E ratio and decrease of E (see Table 1) in accordance with Lind’s claim. Moreover, the tool life increased with increasing coating toughness related to the H^3^/E^2^ ratio, which is a different result than what was presented by Lind et al. [1].

### 3.3. Laboratory Dynamic Impact Test

All of the PVD coatings tested by the dynamic impact test were deposited on the cylindrical HSS substrates. Dynamic impact tests were completed with two loads. The load of 200 N corresponded to the average equivalent pressure ~0.25 GPa and the load of 600 N corresponded to the average equivalent pressure ~0.40 GPa. A series of impacts from 1 up to 500,000 were made, and the size and the volume of the residual impact craters were analyzed.

**Figure 5:**
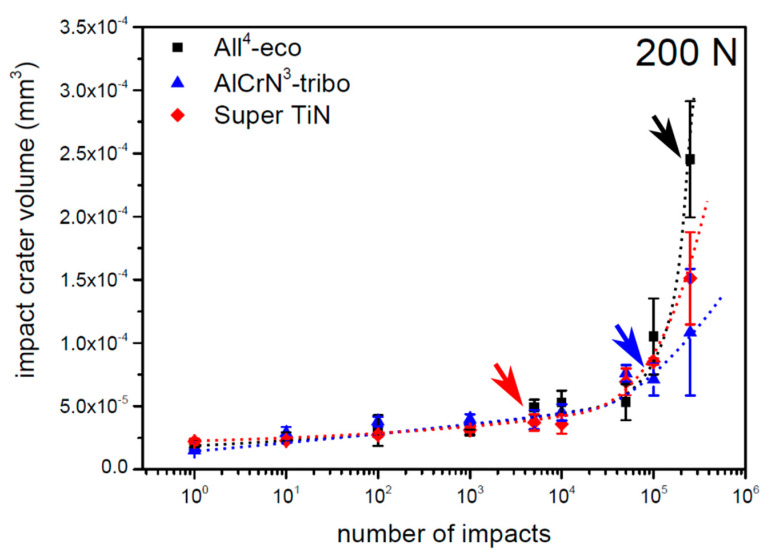
Loading curves of tested PVD coatings obtained by a load of 200 N. Arrows indicates number of impacts where the first cracks appeared.

The basic result of the impact test is a loading curve—the dependence of impact crater volume on the total number of impacts. Generally, as the number of impacts increases from a few impacts, energy supplied from the tester to the coatings is dissipated in the form of elastoplastic deformation of coating and substrate, and the volume of impact craters slowly increases. Part of the supplied energy is accumulated in the form of the stress field in the coating/substrate system, and after reaching a certain value of impacts, stress fields initiate the formation and development of cracks. This cracking leads to coating failure which results in a rapid increase in the volume of impact craters. The highest number of impacts before the coating’s failure is defined as the critical number of impacts (N_C_) [26,38]. Dependence of the impact crater volume on the total number of impacts of all the tested coatings is depicted in Figure 5. All of the tested coatings exhibited N_C_ between 10,000 and 50,000 impacts.

**Figure 6:**
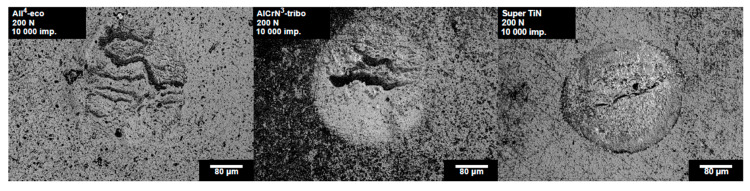
Impact craters of the tested PVD coatings obtained by 10,000 impacts and a load of 200 N.

The impact craters of the tested PVD coatings prepared with a load of 200 N and 10,000 impacts are compared in Figure 6. The impact craters on the coatings All^4^-eco and AlCrN^3^-tribo exhibited smooth edges. Features inside the impact craters are caused by material transport induced by the coating’s deformation. The impact crater of the Super TiN exhibited concentric cracking, indicating an initial phase of the coating failure. The number of impacts corresponding to the first observed cracking is indicated by arrows in Figure 5.

**Figure 7:**
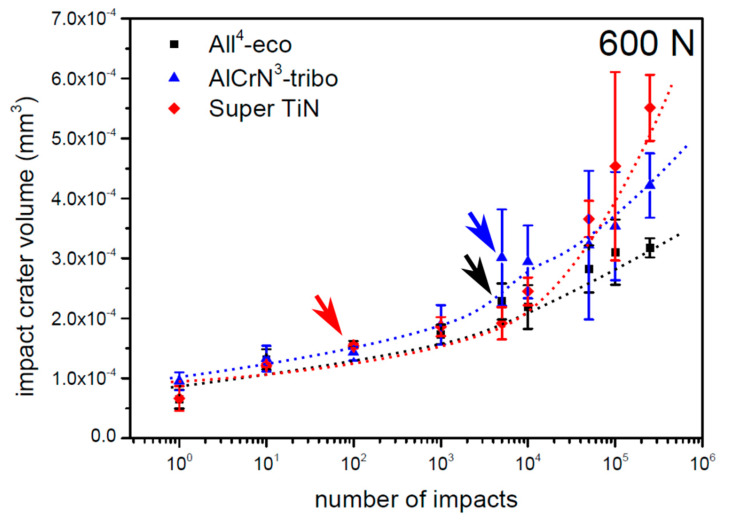
Loading curves of tested PVD coatings obtained by a load of 600 N. Arrows indicates number of impacts where the first cracks appeared.

**Figure 8:**
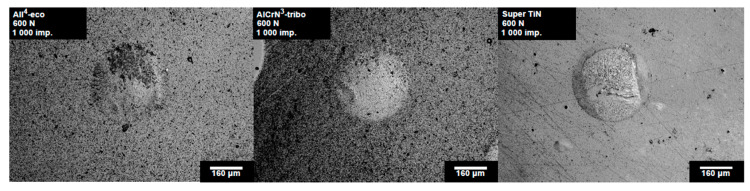
Impact craters of the tested PVD coatings obtained by 1000 impacts and a load of 600 N.

The load curves of the PVD coatings tested with a load of 600 N are depicted in Figure 7. All of the tested coatings exhibited a critical number of impacts between 1000 and 3000 impact cycles. The number of impacts corresponding to the first observation of cracks is in Figure 7 marked with arrows. The impact craters prepared by 1000 impacts with a load of 600 N are depicted in Figure 8. The impact craters on the coatings All^4^-eco and AlCrN^3^-tribo exhibited smooth edges and no features inside the impact craters. However, impact crater of the Super TiN exhibited concentric cracking, which indicated an initial phase of the coating failure, similarly to the case of a load of 200 N.

**Figure 9:**
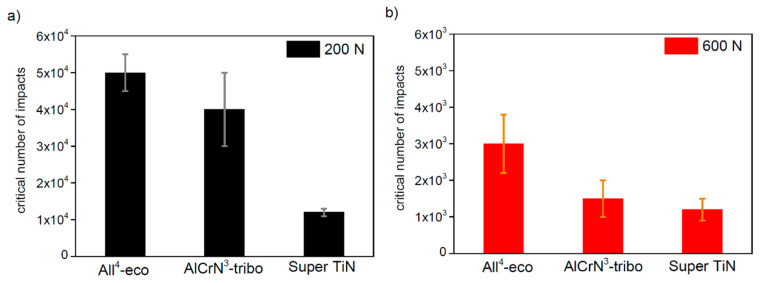
Comparison of the critical number of impacts (N_C_) of the PVD coatings tested by the laboratory dynamic impact test under the impact load of (**a**) 200 N and (**b**) 600 N.

The critical number of impacts was estimated using the comparison of the coating loading curves and by analysis of the impact craters (depth, uncovered crater bottom, cracking). A comparison of the final N_C_ of all of the tested PVD coatings is shown in Figure 9. Results of the impact testing obtained with the impact load of 200 N are compared in Figure 9a, results obtained with the load of 600 N are compared in Figure 9b. The coating All^4^-eco exhibited the highest N_C_ during the impact load of 200 N (50,000) as well as during the impact load of 600 N (3000). On the other hand, the coating Super TiN exhibited the lowest Nc—12,000 impacts at a load of 200 N and 1200 impacts at a load of 600 N.

During impact testing, the whole coating/substrate system is tested [14]. High toughness is important to achieve better resistance against deformation induced by the repeated impact load. As was shown in Table 1, the highest H^3^/E^2^ ratio related to the toughness was exhibited by the coating All^4^-eco, which was coating with the best lifetime under dynamic impact load. Additionally, the first concentric cracking in the case of the All^4^-eco coating was observed at higher numbers of impacts than N_C_. This might indicate a high coating toughness.

On the other hand, the coating with the lowest H^3^/E^2^ ratio, and thus the lowest toughness and resistance to the plastic deformation, was Super TiN—which was the coating with the lowest value of N_C_. Moreover, the Super TiN coating exhibited first concentric cracking at the number of impacts lower than N_C_. Due to the low coating toughness, the first cracking was directly followed by the delamination of the coating on the bottom of the impact crater.

Leyland et al. proposed that the resistance against repeated dynamical impact is related to the H/E ratio [39]. The N_C_ of all of the tested coatings was also related to the H/E ratio and increased with the ratio increase (see Table 1), in accordance with Leyland’s claim.

## 4. Discussion

The tool life of the presented coatings evaluated by the industrial fine blanking process qualitatively correlated with the lifetime presented by N_C_, evaluated by the laboratory dynamic impact test. The coating All^4^-eco exhibited the best tool lifetime and resistance to dynamic impact in both tests. The coating Super TiN exhibited the worst tool lifetime in both tests. The high coating hardness, low effective elastic modulus, and associated high H/E ratio were important both in the industrial fine blanking test and in the laboratory dynamic impact test. Additionally, the ratio of H^3^/E^2^ related to the coating toughness and plastic deformation resistance was related both to the tool life and to the value of N_C_. The Super TiN coating exhibited the best adhesion to the substrate (see Rockwell adhesion and parameter of L_C2_ in Table 2). This demonstrates that the combination of material parameters is, in the case of the industrial fine blanking process and laboratory dynamic impact test, more important than values of particular material parameters.

To obtain the best coating, it is thus necessary to optimize all of those parameters and also other parameters such as residual stress and wear resistance. The procedure of measuring all of those parameters might be time-consuming and thus very disadvantageous for the industry. Moreover, the role of some parameters is still not well specified and clarified, e.g., H^3^/E^2^ ratio. In addition, the determination of coating toughness might be problematic in the case of tough thin films [40]. However, the laboratory impact test includes all of those mechanical and tribological properties and can give direct qualitative results of a lifetime of the set of protective PVD coatings.

The laboratory dynamical impact test seems to be a good option for a qualitative comparison of the behavior of the protective coatings used in the industrial fine blanking process. Moreover, the laboratory impact test is suitable for the pre-testing phase—the selection of the best samples from a huge set of coatings before expensive tests under real industrial conditions. This pre-testing phase is, from an economical point of view, very efficient. Thus, the laboratory impact test can be used for the development of the protective PVD coatings for the industrial fine blanking punches.

## 5. Conclusions

The tool life of the protective PVD coatings in the industrial fine blanking process depends on similar mechanical and tribological properties as in the case of the laboratory dynamic impact test. Moreover, coating failure depends on similar conditions for both methods. This claim was verified by comparing the results of three industrial PVD coatings with different chemical composition and structure, AlCrTi-based nanolayered coating All^4^-eco, AlCr-based nanolayered coating AlCrN^3^-tribo, and Ti-based multi-layered coating Super TiN.

The coatings were prepared using a low-pressure cathodic arc deposition technique and tested by the industrial fine blanking process and by the laboratory dynamic impact test. The lifetime of all of the tested coatings under the dynamic impact test with loads 200 N and 600 N correlated with the tool life of the coatings in the industrial fine blanking process performed under similar pressure. Although the stress distribution during the industrial fine blanking process and laboratory dynamic impact test are different, the laboratory dynamical impact test seems to be a suitable tool for research and development of the protective PVD coatings for punches used in the industrial fine blanking process.

## Figures and Tables

**Table 1:** Mechanical properties and coefficient of friction (CoF) of the tested PVD coatings.

Coating	H (GPa)	E (GPa)	H/E	H^3^/E^2^ (GPa)	CoF
All^4^-eco	41 ± 1	367 ± 6	0.110	0.493	0.9
AlCrN^3^-tribo	39 ± 1	376 ± 8	0.104	0.423	0.4
Super TiN	32 ± 1	388 ± 7	0.082	0.212	1.0

**Table 2:** Adhesion of the tested PVD coatings.

Coating	Rockwell Adhesion	L_C1_ (N)	L_C2_ (N)
All^4^-eco	HF2	53 ± 8	60 ± 7
AlCrN^3^-tribo	HF1	46 ± 7	49 ± 5
Super TiN	HF1	35 ± 5	73 ± 6
